# Functional genomics study of protein inhibitor of activated STAT1 in mouse hippocampal neuronal cells revealed by RNA sequencing

**DOI:** 10.18632/aging.202749

**Published:** 2021-03-24

**Authors:** Kan He, Jian Zhang, Justin Liu, Yandi Cui, Leyna G. Liu, Shoudong Ye, Qian Ban, Ruolan Pan, Dahai Liu

**Affiliations:** 1 Center for Stem Cell and Translational Medicine, School of Life Sciences, Anhui University, Hefei 230601, Anhui, China; 2 Department of Biostatistics, School of Life Sciences, Anhui University, Hefei 230601, Anhui, China; 3 Department of Statistics, University of California, Riverside, CA 92521, USA; 4 Portola High School, Irvine, CA 92618, USA; 5 Foshan Stomatology Hospital, School of Medicine, Foshan University, Foshan 528000, Guangdong, China

**Keywords:** PIAS1, HT-22, RNA-seq, pathway, AD

## Abstract

Protein inhibitor of activated STAT1 (PIAS1), a small ubiquitin-like modifier (SUMO) E3 ligase, was considered to be an inhibitor of STAT1 by inhibiting the DNA-binding activity of STAT1 and blocking STAT1-mediated gene transcription in response to cytokine stimulation. PIAS1 has been determined to be involved in modulating several biological processes such as cell proliferation, DNA damage responses, and inflammatory responses, both *in vivo* and *in vitro*. However, the role played by PIAS1 in regulating neurodegenerative diseases, including Alzheimer’s disease (AD), has not been determined. In our study, significantly different expression levels of PIAS1 between normal controls and AD patients were detected in four regions of the human brain. Based on a functional analysis of *Pias1* in undifferentiated mouse hippocampal neuronal HT-22 cells, we observed that the expression levels of several AD marker genes could be inhibited by *Pias1* overexpression. Moreover, the proliferation ability of HT-22 cells could be promoted by the overexpression of *Pias1*. Furthermore, we performed RNA sequencing (RNA-seq) to evaluate and quantify the gene expression profiles in response to *Pias1* overexpression in HT-22 cells. As a result, 285 significantly dysregulated genes, including 79 upregulated genes and 206 downregulated genes, were identified by the comparison of *Pias1*/+ cells with WT cells. Among these genes, five overlapping genes, including early growth response 1 (*Egr1*), early growth response 2 (*Egr2*), early growth response 3 (*Egr3*), FBJ osteosarcoma oncogene (*Fos*) and fos-like antigen 1 (*Fosl1*), were identified by comparison of the transcription factor binding site (TFBS) prediction results for STAT1, whose expression was evaluated by qPCR. Three cell cycle inhibitors, p53, p18 and p21, were significantly downregulated with the overexpression of *Pias1*. Analysis of functional enrichment and expression levels showed that basic region leucine zipper domain-containing transcription factors including zinc finger C2H2 (zf-C2H2), homeobox and basic/helix-loop-helix (bHLH) in several signaling pathways were significantly involved in PIAS1 regulation in HT-22 cells. A reconstructed regulatory network under PIAS1 overexpression demonstrated that there were 43 related proteins, notably Nr3c2, that directly interacted with PIAS1.

## INTRODUCTION

PIAS1, named as protein inhibitor of the activated STAT1, which is one of E3-type small ubiquitin-like modifier (SUMO) ligases and plays important roles in diverse cellular pathways, such as the STAT, p53 and steroid hormone signaling pathways. Based on the results of *in vivo* and *in vitro* experiments, PIAS1 was reported to regulate innate immune responses mediated by the interferon (IFN)-gamma- or -beta-inducible genes by inhibiting the STAT pathway [1]. The STAT1 protein is an essential transcription factor functioning in inflammatory activation in Alzheimer’s disease (AD) brains [2–5]. PIAS1 may function as an E3 ligase for ligand-activated nuclear receptor peroxisome proliferator-activated receptor (PPAR) gamma, which is modified by SUMO-1 and is a potential target for apoptosis-induction therapy in cancer cells [6]. Based on a previous study of *Pias1*-/- cells that employed microarray analysis, PIAS1 was also identified as a novel negative regulator of NF-κB that is associated with immunity and homeostasis in a number of human illnesses, such as chronic inflammatory diseases and cancer [7–9]. PIAS1 acts as one of the positive transcriptional coregulators of GATA-3, and its molecular interaction is reported to be important for Th2 immune responses by regulating IL-13 [10]. Two members of the PIAS family proteins, PIAS1 and PIAS3, were demonstrated to interact with TATA-binding protein (TBP) [11]. It has been showed that PIAS1 associates with protein-tyrosine phosphatase 1B (PTP1B) in mammalian fibroblasts and catalyzes sumoylation of PTP1B, thereby regulating metabolism and cell proliferation [12]. PIAS1 can promote the proliferation of fibroblasts, smooth muscle cells, and intestinal epithelial cells by interacting with the Kruppel-like factor 5 (KLF5) and enhancing its ability [13]. PIAS1 was reported to confer DNA-binding specificity to the homeoprotein Msx1 in regulating myoblast differentiation through repression of the myogenic regulatory gene MyoD [14]. An epigenetic mechanism has been elucidated that PIAS1 might recruit DNA methyltransferases and heterochromatin protein 1 by binding to the Foxp3 promoter during the natural regulatory T cell differentiation [15]. It is reported that the suppression of PIAS1 abolished the ability of arsenic trioxide, an effective treatment of acute promyelocytic leukemia (APL), to trigger apoptosis in APL cells [16]. During the adipogenesis, PIAS1 plays a dynamic role by promoting the SUMOylation of CCAAT/enhancer-binding protein beta (C/EBPbeta) [17]. The epigenetic pathway associated with PIAS1 may play important roles in regulating the hematopoietic stem cells self-renewal and differentiation [18]. A critical function of PIAS1 in controlling insulin sensitivity by inhibiting the inflammatory cascade has also been observed in adipose tissue [19]. The phosphorylation of PIAS1 was reported to mediate SUMOylation of Elk-1, which may function in promoting neuronal survival in APP/PS1 mice [20].

PIAS1 may serve to regulate the accumulation of Huntingtin (HTT) proteins and its modulation in neurons, which can alter Huntington's disease (HD)-associated phenotypes *in vivo* [21, 22]. However, the pathological and molecular functions of PIAS1 in other neurodegenerative disorders has not been studied, especially for AD. Therefore, it is particularly important to explore the function of PIAS1 in AD and analyze its molecular regulation mechanism.

## RESULTS AND DISCUSSION

### Expression and regulation of PIAS1 in AD and HT-22 cells

**Figure 1:**
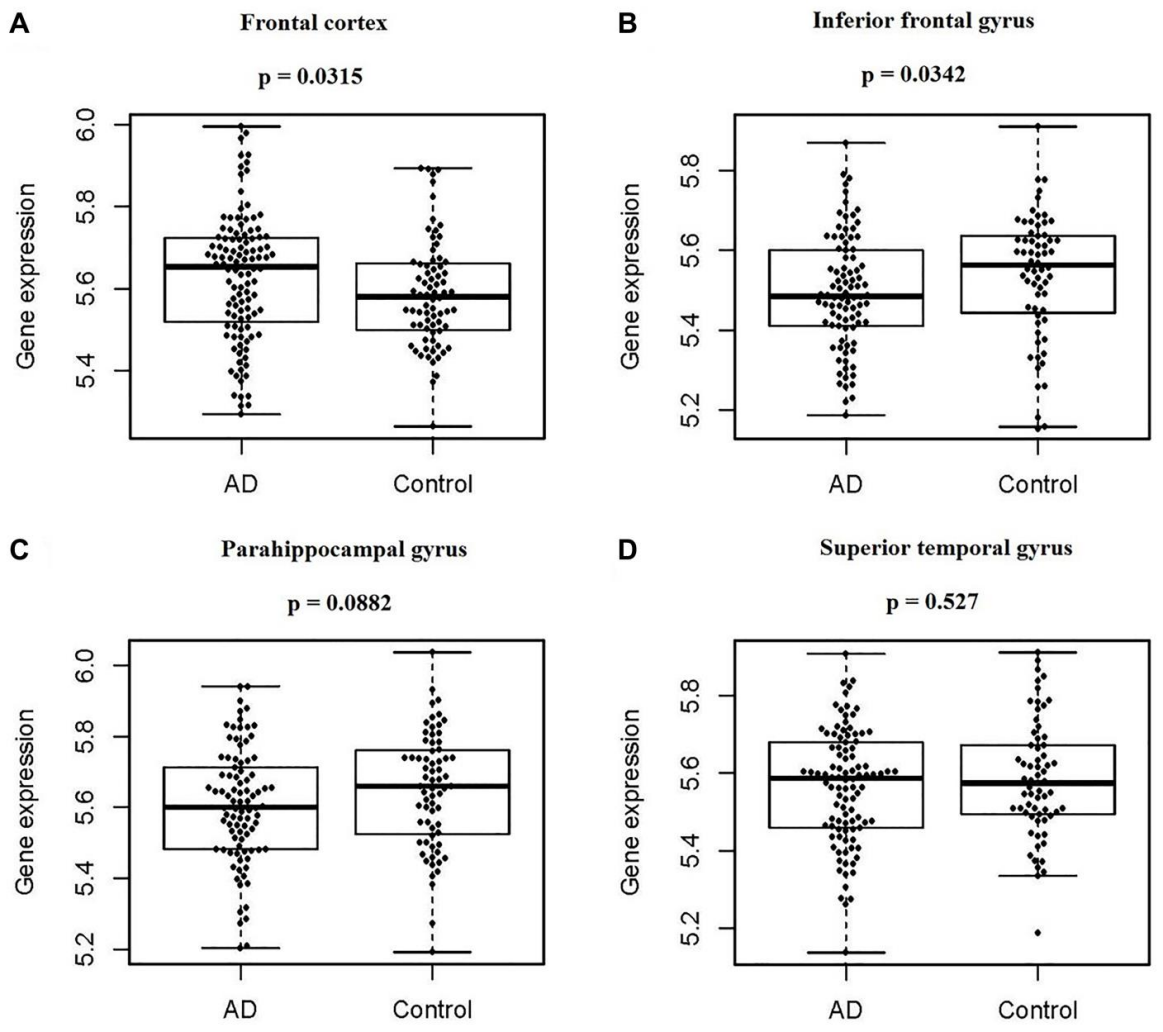
**PIAS1 gene expression in four regions of human brains between normal controls and AD patients.** Based on the Synapse database, the difference in PIAS1 gene expression in four regions of human brains between normal controls and AD patients was determined by using the Wilcoxon signed-rank test. (**A**) For the frontal cortex, there were 111 cases and 76 controls, and PIAS expression was significantly higher in AD patients, with a *p* value of 0.0315. (**B**) For the inferior frontal gyrus, there were 90 cases and 64 controls, and PIAS expression was significantly lower in AD patients, with a *p* value of 0.0342. (**C**) For the parahippocampal gyrus, there were 90 cases and 68 controls, and PIAS expression was not significantly changed, with a *p* value of 0.0882. (**D**) For the superior temporal gyrus, there were 102 cases and 65 controls, and PIAS expression was not significantly changed, with a *p* value of 0.527.

Based on analyzing the differences of the PIAS1 gene expression levels in four human brain regions between the normal controls and AD patients, the expression of PIAS1 was found to be lower in both the inferior frontal gyrus (*p* = 0.0342) and parahippocampal gyrus (*p* = 0.0882) in AD samples. In the frontal cortex, the expression of PIAS1 was detected to be significantly higher (*p* = 0.0315) in AD samples. No significant difference was identified between the control and AD samples in the superior temporal gyrus. These results are presented in Figure 1.

**Figure 2:**
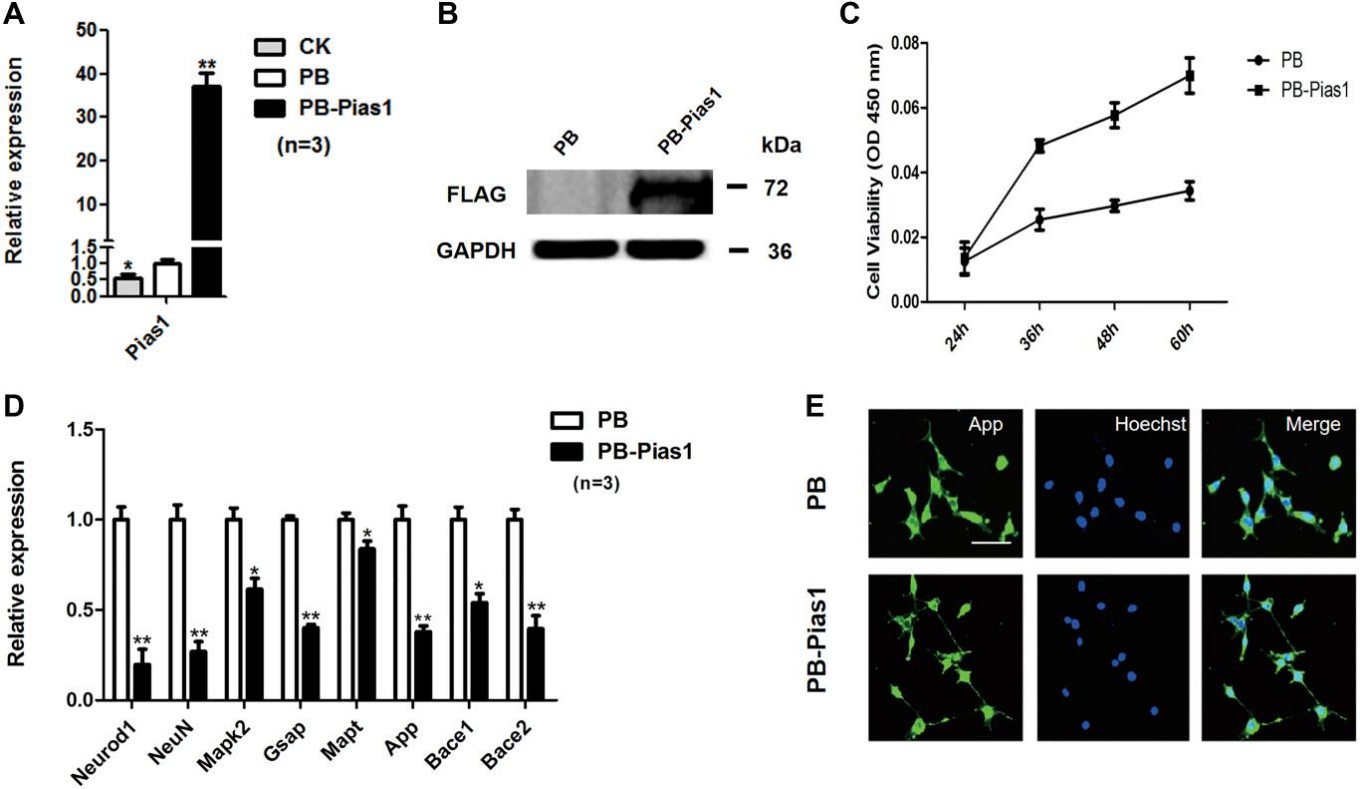
**Overexpression of *Pias1* regulates AD marker gene expression and cell proliferation in HT-22 cells.** (**A**) qRT-PCR analysis of *Pias1* mRNA expression in the hippocampal neuronal HT22 cells transfected with the indicated transgenes. Data represent the mean ± SD of three biological replicates. ^*^p < 0.05, ^**^p < 0.01 versus PB vector. The control check (CK) group represents the baseline expression of Pias1 in HT-22 cells. (**B**) Western blot analysis of PIAS1 in the hippocampal neuronal HT22 cells with stable *Pias1* transgenic expression. (**C**) Cell proliferation was determined by CCK-8 assays in HT-22 cells transfected with PiggyBac vector (PB) or PB-Pias1. Data represent the mean ± SD. ^*^p < 0.05, ^**^p < 0.01. (**D**) qRT-PCR analysis of *Neurod1, NeuN, Mapk2, Gsap, Mapt, App, Bace1* and *Bace2* expression levels in PB and PB-*Pias1* hippocampal neuronal HT22 cells. Data are presented as the mean ± SD of three independent experiments. ^*^p < 0.05, ^**^p < 0.01. (**E**) Immunofluorescence staining of APP in the hippocampal neuronal HT22 cells overexpressing PB-*Pias1*. Bar: 100 μm.

By transfecting the recombinant plasmid, the stable HT-22 cells under the overexpression of *Pias1* (*Pias1/+*) was established. The qRT-PCR results showed that through the over expression experiment, we have significantly increased the expression of *Pias1* (Figure 2A). According to the results of Western blotting (WB), the protein expression of PIAS1 was also found to be higher in *Pias1*/+ cells that is labeled as ~ 72 kDa protein (Figure 2B). The CCK-8 assay results showed that the proliferation of HT-22 cells was enhanced in *Pias1*/+ cells (Figure 2C). The neurite outgrowth of HT-22 cells could also be enhanced by the overexpression of *Pias1*. In a functional study of PIAS1 in the regulation of cell proliferation in human prostate cancer (HPC), PIAS1 was reported to be increased in HPC and to enhance cell proliferation through inhibition of cyclin-dependent kinase inhibitor 1A (P21) [23]. The activation of the PIAS1-modulated Smad2/4 complex was demonstrated to mediate zinc-induced apoptosis of prostate cancer cells [24]. PIAS1 has promotive effects on cell proliferation by upregulating the activity of KLF5 in various cell types [13].

To explore the functional roles of PIAS1 in AD, some marker genes such as neuron-specific nuclear proteins including neuronal differentiation 1 (*Neurod1*) and *NeuN* encoded by FOX3, mitogen-activated protein kinase 2 (*Mapk2*), gamma-secretase activating protein (*Gsap*), microtubule associated protein tau (*Mapt*), amyloid beta precursor protein (*App*), beta-site APP-cleaving enzyme 1 (*Bace1*) and beta-site APP-cleaving enzyme 2 (*Bace2*) were investigated to determine their expression changes in response to overexpression of *Pias1*. All of these genes expressions were shown to be significantly decreased in the *Pias1*/+ group (Figure 2D). Moreover, according to the immunofluorescence analysis, it was showed that the axonal outgrowth of HT-22 cells could be increased by the overexpression of *Pias1* (Figure 2E). These results suggested that *Pias1* might have potential functions in the regulation of AD and neuronal development.

### Differentially expressed genes analysis in response to *Pias1* overexpression in HT-22 cells

**Figure 3:**
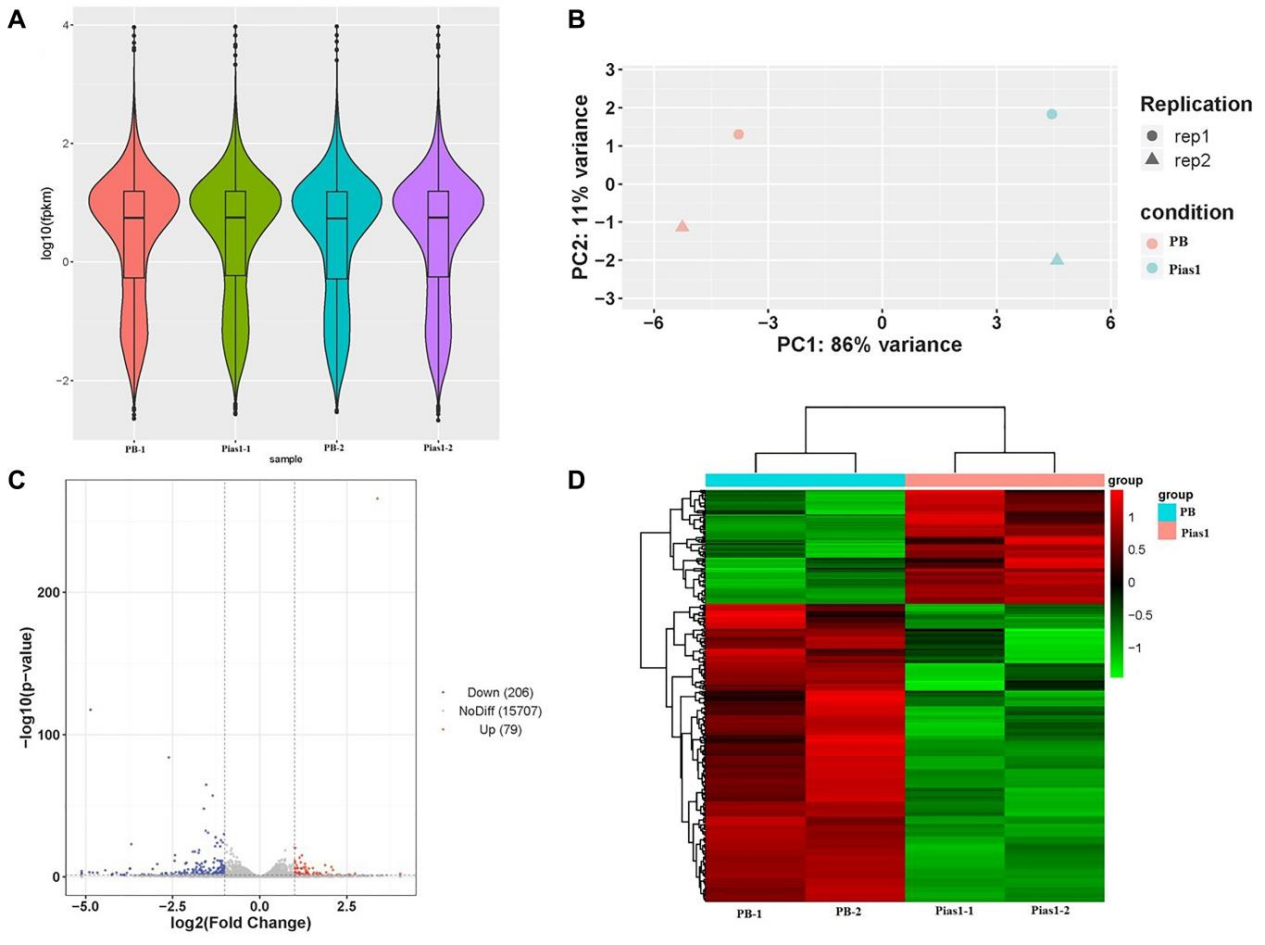
**RNA-seq data quality and DEG analysis.** (**A**) The violin plot based on values of log10 (fpkm) showed the whole gene expression distribution of four groups, including PB-1, PB-2, Pias1-1 and Pias1-2. (**B**) The PCA plot showed how well the four sequencing samples (PB-1, PB-2, Pias1-1 and Pias1-2) are separated from each other. (**C**) The scatter plot showed the results of DEGs based on the values of log2 (fold change) and -log10 (*p* value), including 206 downregulated genes, 79 upregulated genes and 15707 genes with no significant differences. (**D**) The heatmap showed the expression patterns of 285 significantly dysregulated genes with FC greater than 2 and *p* value less than 0.05, including 79 upregulated genes and 206 downregulated genes, by comparison of the *Pias1*/+ group to the WT group.

To screen the downstream target genes in HT-22 cells by overexpressing *Pias1*, we performed RNA-seq to obtain the transcriptome data of two cell types, WT cells and *Pias1*/+ cells. The sequencing data quality is shown in Table 1. Our data were of high quality, exhibiting a Q20 value greater than 95% and a Q30 value greater than 90%. Based on the data preprocessing and transcriptome assembly, a total of 21,862 annotated transcripts were identified in this study. According to the differentially expressed genes (DEGs) analysis, there were 285 significantly dysregulated genes, including 79 upregulated genes (FC > 2, *p* < 0.05) and 206 downregulated genes (FC < 1/2, *p* < 0.05), as determined by the comparison of the *Pias1*/+ group to the WT group. The details of the up- and downregulated DEGs are shown in Supplementary Table 1. The PCA of sequencing samples, as well as the distribution and clustering of DEGs, are shown in Figure 3.

**Table 1.** The quality control of RNA-seq data.

**Sample**	**Raw reads**	**Clean reads**	**Clean reads %**	**Q20 %**	**Q30 %**
PB-1	48213112	45042086	93.42	97.38	92.97
Pias1-1	45822220	42887740	93.59	97.36	92.89
PB-2	50567794	47369520	93.67	97.27	92.71
Pias1-2	48265814	45448864	94.16	97.40	92.97

**Figure 4:**
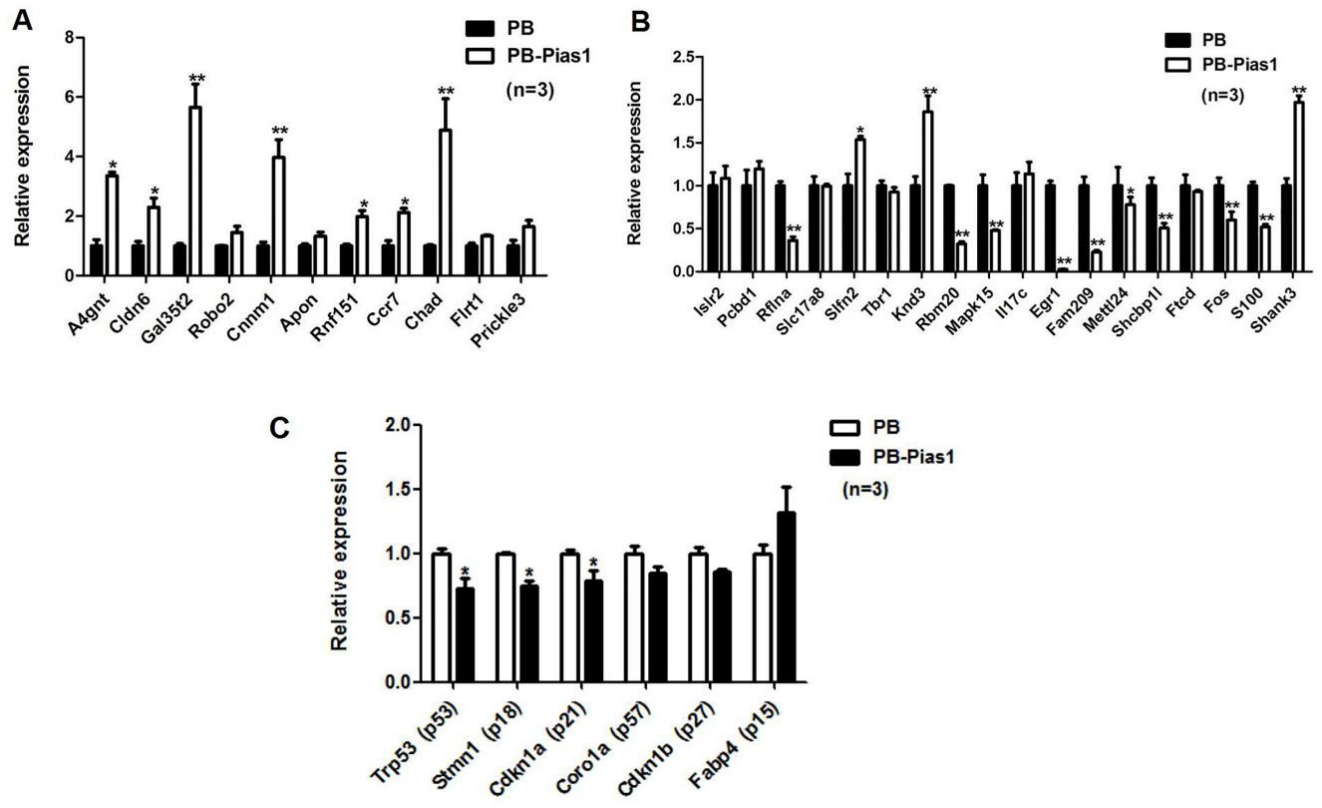
**Validation of target gene expression by qRT-PCR.** (**A**) For upregulated DEGs, 11 genes were selected for the following qRT-PCR validation. (**B**) For downregulated DEGs, 18 genes were selected for the following qRT-PCR validation. (**C**) The PIAS1 regulation of cell cycle inhibitors, including p15, p18, p21, p27, p53 and p57, was tested by qRT-PCR. Among them, three inhibitors, p53, p18 and p21, were significantly downregulated under the overexpression of *Pias1*. ^*^p < 0.05, ^**^p < 0.01.

To test the accuracy of screening DEGs by high-throughput sequencing, we selected 11 upregulated genes and 18 downregulated genes for the following qRT-PCR validation. The results showed that 7 out of 11 upregulated genes were significantly identified in the *Pias1*/+ group, they were *A4gnt, Cldn6, Gal3st2, Cnnm1, Rnf151, Ccr7* and *Chad* (Figure 4A). Human alpha1,4-N-acetylglucosaminyltransferase (*A4gnt*) was reported to function in O-glycan biosynthesis [25]. The expression of Claudin-6 (*Cldn6*) in early development was reported to be relevant to definitive endoderm derivatives [26]. In addition, 9 out of 18 downregulated genes were significantly identified in the *Pias1*/+ group, they were *Rflna, Rbm20, Mapk15, Egr1, Fam209*, *Mettl24, Shcbp1l, Fos* and *S100b* (Figure 4B). We further examined the PIAS1 regulation of cell cycle inhibitors, including p15, p18, p21, p27, p53 and p57. Among these genes, three inhibitors, p53, p18 and p21, were significantly downregulated with the overexpression of Pias1 (Figure 4C).

### Signaling pathway and gene ontology enrichment under the overexpression of *Pias1* in HT-22 cells

**Figure 5:**
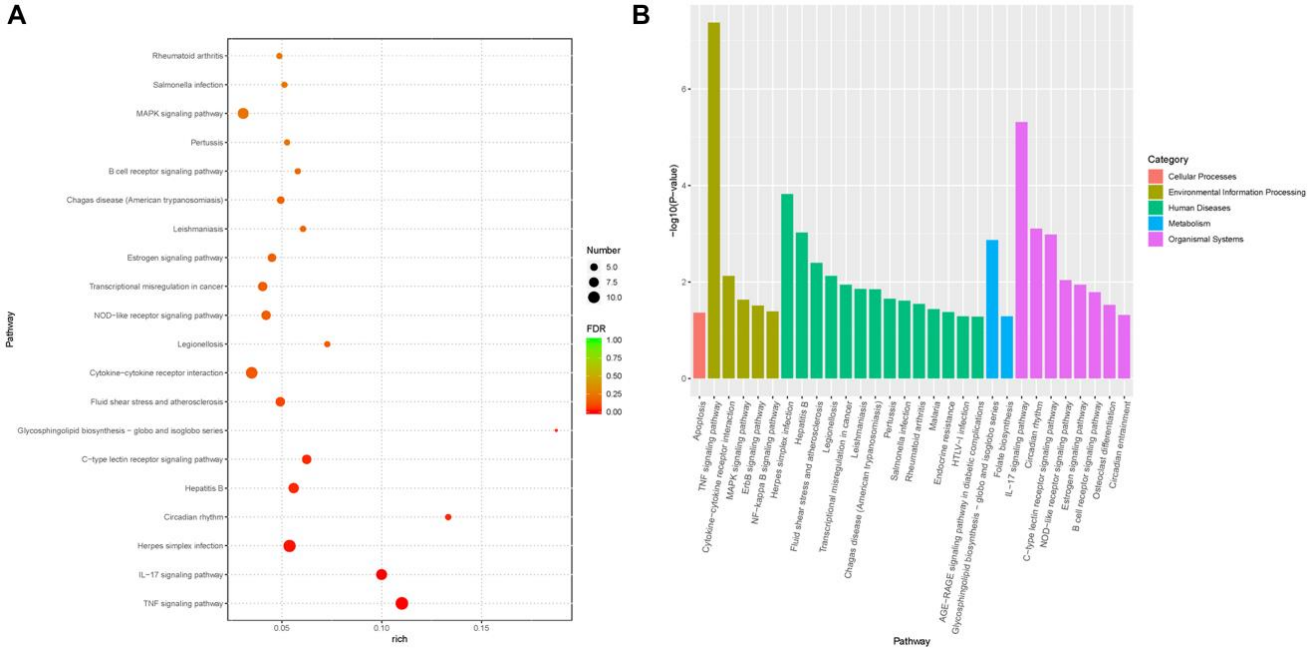
**Functional enrichment analysis of DEGs from RNA-seq data.** (**A**) The plot showed the top 20 significantly enriched KEGG pathways of differentially expressed genes (DEGs) associated with *Pias1* regulation with an FDR value cutoff of 0.05. Round size represents the gene count of each pathway, and the color represents the significance level. (**B**) The bar chart showed the categories of significantly enriched pathways. There were five pathway categories including cellular processes (red), environmental information processing (green yellow), human diseases (green), metabolism (blue) and organismal systems (purple).

Based on GSEA, 30 significantly enriched KEGG pathways were identified to be involved in the regulation of *Pias1* in HT-22 cells, which are shown in Figure 5. Among these pathways, the top two significantly enriched pathways were the TNF signaling pathway (FDR=4.25E–08) in terms of environmental information processing and the IL-17 signaling pathway (FDR=4.88E–06) in terms of organismal systems. In addition, other signaling pathways, including the MAPK signaling pathway (FDR=2.34E–02), the ErbB signaling pathway (FDR=3.09E–02) and the NF-kappa B signaling pathway (FDR=4.11E–02), were also identified as being significantly associated with *Pias1* regulation. The genes involved in each pathway are shown in Table 2.

**Table 2.** Significantly enriched signaling pathways and dysregulated genes.

**Pathway**	**FDR**	**Up_genes**	**Down_genes**
TNF signaling pathway	4.25E–08	–	Fas, Creb5, Gm5431, Jun, Lif, Junb, Fos, Ptgs2, Cxcl1, Vcam1, Ccl2, Mmp9
IL-17 signaling pathway	4.88E–06	–	Mapk15, Fosl1, Il17c, Jun, Fos, Ptgs2, Cxcl1, Ccl2, Mmp9
MAPK signaling pathway	2.34E–02	Hspa1l	Fas, Jun, Dusp6, Dusp1, Nr4a1, Fos, Areg, Epha2
ErbB signaling pathway	3.09E–02	–	Jun, Nrg1, Areg, Hbegf
NF-kappa B signaling pathway	4.11E–02	Blnk	Ptgs2, Vcam1, Plau

Moreover, we have also identified several significantly enriched GO terms, which are listed in Supplementary Table 2. The top three biological process (BP) terms were response to lipid (GO:0033993), regulation of cell population proliferation (GO:0042127) and response to steroid hormone (GO:0048545). The molecular function (MF) terms were primarily related to DNA-binding transcription factor activity, steroid hormone receptor activity and transcription regulator activity. The cellular component (CC) term of the transcription factor AP-1 complex (GO:0035976) was significantly enriched with the target genes *Fos, Jun* and *Junb*.

### Transcriptional regulation of genes related to the overexpression of *Pias1* in HT-22 cells

**Figure 6:**
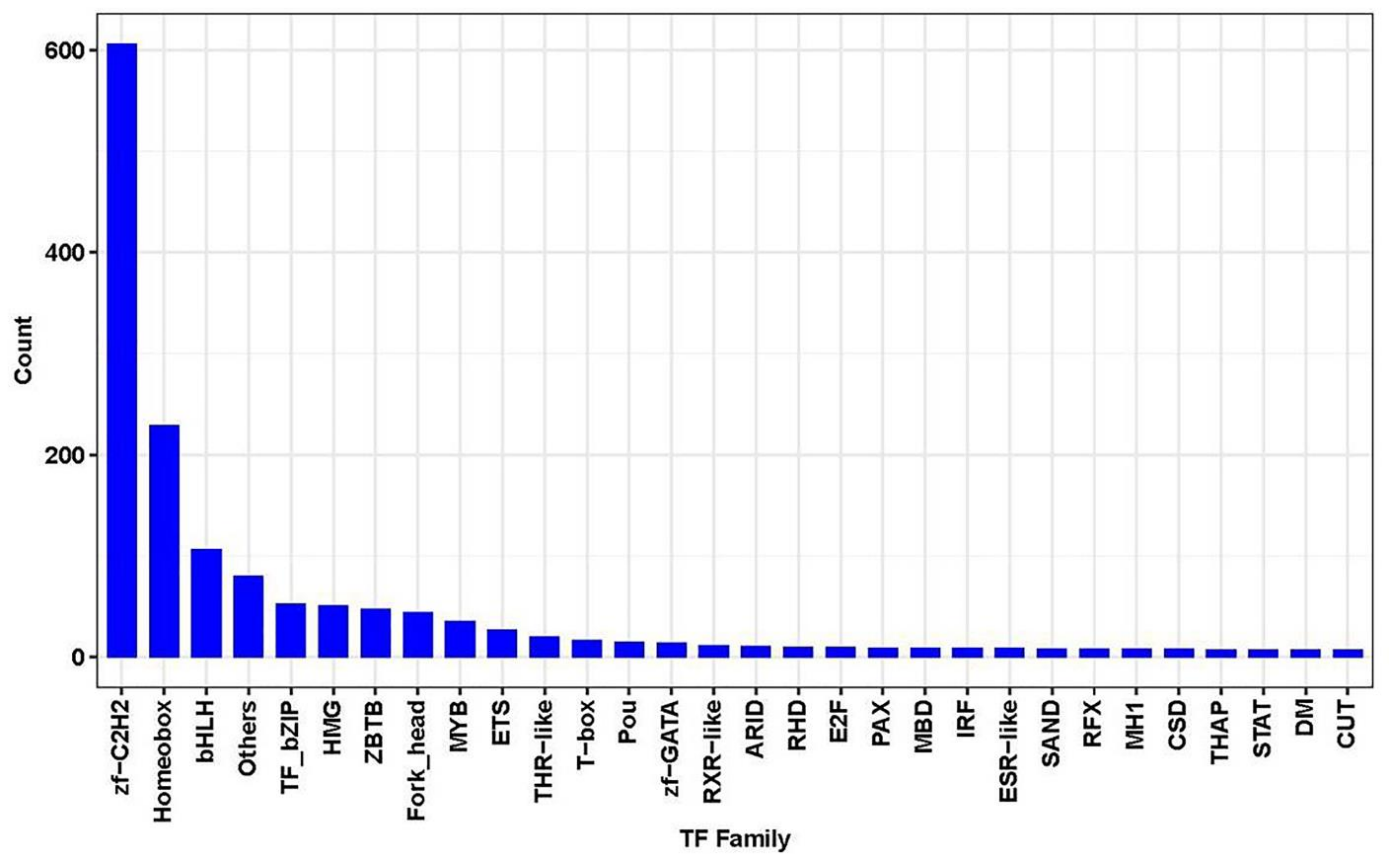
**Results of TF family gene counts.** This chart showed the top significantly enriched TF family (X-axis) with the gene count (Y-axis).

To further investigate transcription factor (TF) gene regulation associated with the overexpression of *Pias1* in HT-22 cells, we analyzed the enrichment of transcription factors regulating target DEGs (Figure 6). As a result, the most strongly enriched TFs were zinc finger C2H2 (zf-C2H2), homeobox and basic/helix-loop-helix (bHLH) proteins. More than 100 genes were encoded or regulated by these three enriched TFs. Using the SMART tool to analyze the proteins with the same domain composition that have at least one copy of each of the domains of the query, we identified the significant domains associated with target proteins. The results are shown in Table 3. The first domain was the basic region leucine zipper (FDR=1.00E–03) with the target factors Creb5, Dbp, Fos, Fosl1, Jun, Junb and Tef. The second domain was the leucine-rich repeat C-terminal domain (FDR=1.60E–03) with the target factors Chad, Flrt1, Gp1ba, Igsf10, Islr, Islr2, Lrrc19 and Tlr2. The third domain was tumor necrosis factor receptor or nerve growth factor receptor repeats (FDR=1.60E–03) with the target factors Eda2r, Fas, Tnfrsf14, Tnfrsf18 and Tnfrsf26.

**Table 3.** SMART protein domains.

**Domains**	**FDR**	**Target proteins**
basic region leucin zipper	1.00E–03	Creb5, Dbp, Fos, Fosl1, Jun, Junb, Tef
Leucine rich repeat C-terminal domain	1.60E–03	Chad, Flrt1, Gp1ba, Igsf10, Islr, Islr2, Lrrc19, Tlr2
Tumor necrosis factor receptor / nerve growth factor receptor repeats	1.60E–03	Eda2r, Fas, Tnfrsf14, Tnfrsf18, Tnfrsf26
PAS domain	1.70E–03	Arnt2, Pde8a, Per1, Per2, Per3
Epidermal growth factor-like domain	1.70E–03	Adam33, Areg, Celsr1, Cspg5, Gpr179, Hbegf, Nrg1, Plau, Ptgs2, Susd1, Tnfrsf18
c4 zinc finger in nuclear hormone receptors	1.70E–03	Nr1d1, Nr1d2, Nr1h4, Nr3c2, Nr4a1, Nr6a1
Ligand binding domain of hormone receptors	1.70E–03	Nr1d1, Nr1d2, Nr1h4, Nr3c2, Nr4a1, Nr6a1
Leucine rich repeat N-terminal domain	5.20E–03	Amigo3, Chad, Flrt1, Gp1ba, Igsf10, Islr, Islr2
Leucine-rich repeats, typical (most populated) subfamily	5.40E–03	Amigo3, Chad, Flrt1, Gp1ba, Igsf10, Islr, Islr2, Tlr2
Motif C-terminal to PAS motifs (likely to contribute to PAS structural domain)	6.70E–03	Arnt2, Per1, Per2, Per3
Leucine-rich repeats, outliers	9.40E–03	Amigo3, Chad, Flrt1, Gp1ba, Igsf10, Islr, Islr2, Lrrc19, Lrrc73, Tlr2

**Figure 7:**
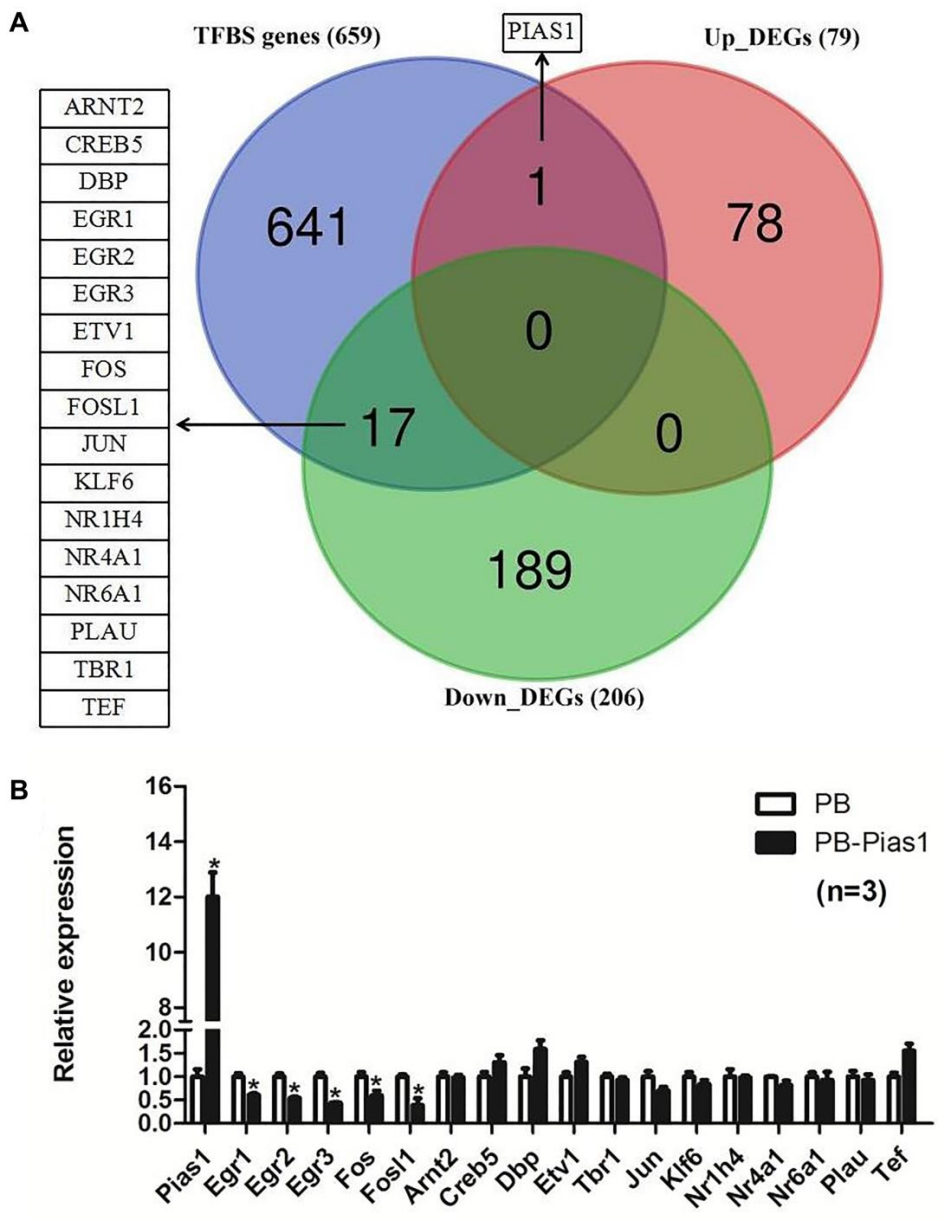
**Overlapping targets between transcription factor binding site genes of STAT1 and DEGs of PIAS1.** (**A**) Transcription factor binding site (TFBS) prediction for STAT1 was performed and compared with the differentially expressed genes of RNA-seq results in PIAS1 overexpression cell lines. As a result, 18 overlapping genes, including one upregulated gene PIAS1 and 17 downregulated genes, were identified as the target genes. (**B**) The regulation of the overlapping genes was evaluated by qPCR. As a result, five genes, including early growth response 1 (*Egr1*), early growth response 2 (*Egr2*), early growth response 3 (*Egr3*), FBJ osteosarcoma oncogene (*Fos*) and fos-like antigen 1 (*Fosl1*), were significantly downregulated in PIAS1-overexpressing cells.

According to the transcription factor binding site (TFBS) prediction, we identified 659 TFBS genes for STAT1. Based on the comparison of 206 downregulated DEGs and 79 upregulated DEGs by overexpressing PIAS1, there were 18 overlapping genes, including one upregulated gene, PIAS1, and 17 downregulated genes. The details of this analysis are shown in Figure 7A. The expression levels of five TFBS genes were identified to be significantly decreased in response to PIAS1 overexpression. They were early growth response 1 (*Egr1*), early growth response 2 (*Egr2*), early growth response 3 (*Egr3*), FBJ osteosarcoma oncogene (*Fos*) and fos-like antigen 1 (*Fosl1*) (Figure 7B). The immediate early gene *Egr1* (also known as *Zif268*) is required for the maintenance of late long-term potentiation (LTP) in the dentate gyrus of the hippocampus and for the expression of long-term memory [27]. *Egr1* was found to have functions in the activation of β-secretase 1 (BACE–1) and acceleration of amyloid-β peptide (Aβ) synthesis in the AD patients brains, which was suggested as one of potential therapeutic candidates for the treatment of AD [28, 29]. During long-term memory formation in the context of the preexposure facilitation effect (CPFE), both *Egr1* and *Fos* (also known as *c-Fos*) have been identified as differentially expressed immediate early genes [30]. *Egr2* has been reported to be a downstream target of NF-κB in neurons, which plays an important role in peripheral nervous system myelination [31, 32]. Not completely consistent with *Egr1*, the role of *Egr3* in learning and memory processing has also been revealed [33]. *Fosl1* is an immediate-early gene that has been identified as a target of the NMDAR-Wnt/β-catenin signaling pathway in the hippocampus [34, 35].

### Protein-protein interaction network construction in response to *Pias1* overexpression

**Figure 8:**
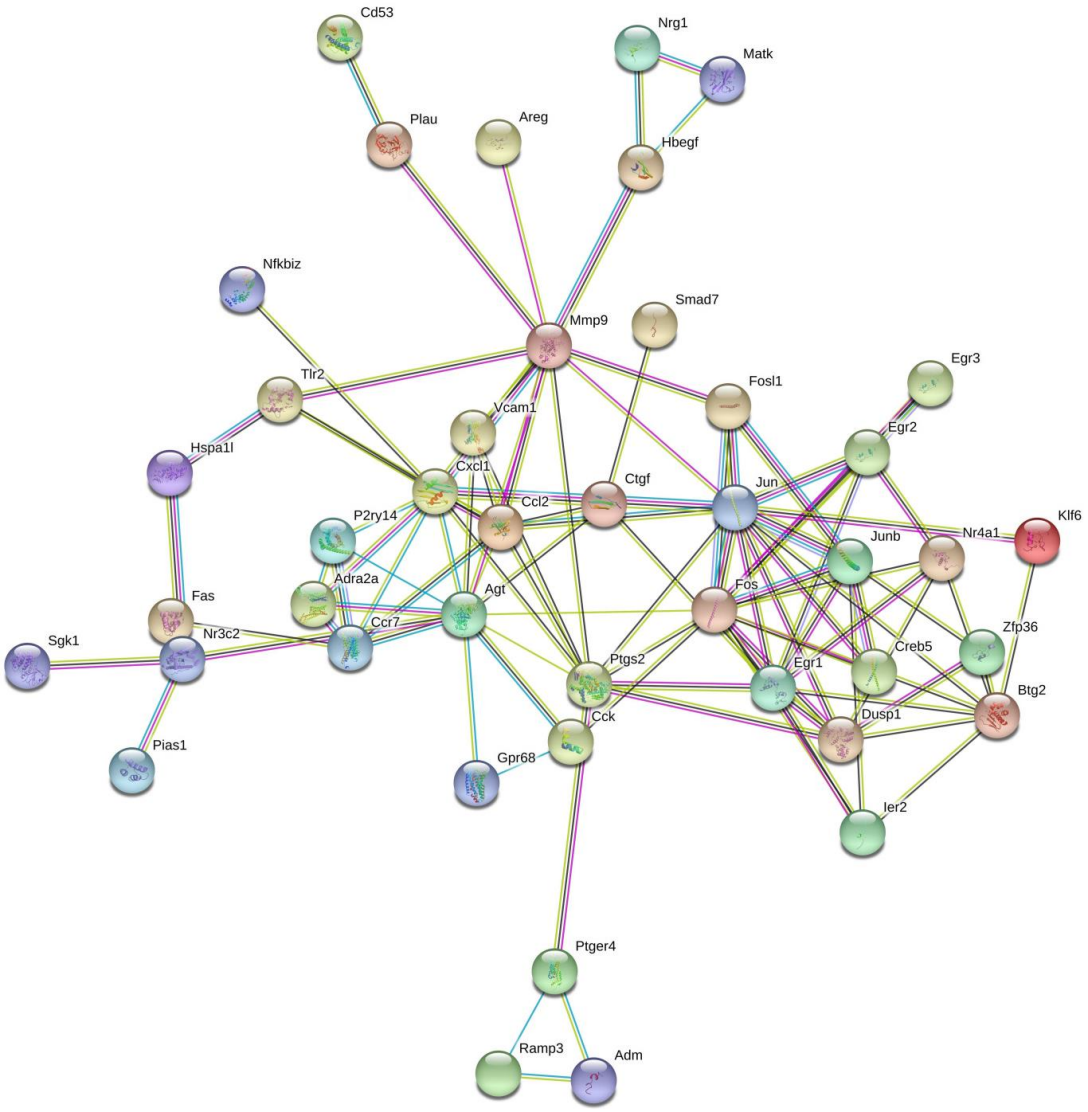
**Protein-protein interaction network associated with *Pias1* overexpression in HT-22 cells.** The protein-protein interaction network was reconstructed by the top 43 DEG proteins in response to *Pias1* overexpression in HT-22 cells, such as Nr3c2.

In addition, a regulatory network of protein-protein interaction in response to the overexpression of *Pias1* in HT-22 cells was constructed (Figure 8). A total of 43 encoding DEGs were involved in this network. Our findings provide evidence for a direct link between PIAS1 and NR3C2. The *Nr3c2* gene encodes the mineralocorticoid receptor (MLR), which plays important roles in blood pressure control and fluid balance by regulating the amount of sodium in the body [36]. MLR has been determined to be involved in guiding spatial and stimulus-response learning in mice [37]. In humans, PIAS1 was reported to repress MLR ligand-dependent transcriptional activity through the interaction with the N-terminal domain of MLR through SUMO modifications [38]. The upstream gene was *Agt* (angiotensinogen), acting as a serpin peptidase inhibitor, which is important for the renin-angiotensin system (RAS) [39]. The pathophysiology and morphology of RAS in mice brains may be influenced by the knockout of neurolysin [40]. The cell death and production of reactive oxygen species (ROS) in the mouse brain may be restrained by the ACE2/Ang-(1-7)/Mas pathway activation with angiotensin II overproduction [41].

In conclusion, the regulatory mechanism of the *Pias1* gene in MHNC was elucidated in our functional genomics study by using RNA-seq. The overexpression of *Pias1* was determined to repress several AD markers expressions and to accelerate the proliferation of HT-22 cells. There were 79 upregulated genes and 206 downregulated genes, significantly identified in *Pias1*/+ cells compared to WT cells through the DEGs analysis. Functional enrichment analysis and TFBS prediction results showed that basic region leucine zipper domain-containing transcription factors including zinc finger C2H2 (zf-C2H2), homeobox and basic/helix-loop-helix (bHLH) in several signaling pathways were significantly involved in PIAS1 regulation in MHNCs. We also reconstructed the regulatory network under PIAS1 overexpression with 43 coding proteins, especially NR3C2, which directly interacted with PIAS1.

## MATERIALS AND METHODS

### PIAS1 gene expression in AD brains

The expression profiles of the PIAS1 gene in four brain regions in humans, including the frontal pole (including 111 AD patients and 76 controls), the inferior frontal gyrus (including 90 AD patients and 64 controls), the parahippocampal gyrus (including 90 AD patients and 68 controls), and the superior temporal gyrus (including 102 AD patients and 65 controls), were sourced from the database Synapse [42, 43]. Next, we tested the differences of PIAS1 gene expressions between AD patients and controls in each brain region. The Wilcoxon signed-rank test was used to analyze the significance.

### Cell culture and gene overexpression

For the *in vitro* experiments, we employed one subclone of the mouse hippocampal HT-22 cells, which were purchased from CHI SCIENTIFIC, Shanghai, China. HT-22 cells were maintained in Dulbecco’s modified Eagle’s medium (DMEM, Sigma) supplemented with 10% fetal bovine serum (FBS, Gibco BRL, USA), MEM nonessential amino acids (Invitrogen), L-glutamine (Invitrogen), penicillin and streptomycin (Sigma), 0.01 mM β-mercaptoethanol (Invitrogen) and 1000 units/ml LIF (Millipore). Based on the database of GenBank, the reference sequence of Mus musculus *Pias1* (NM_019663.3) was obtained. The gene insertion and transfection were performed by using a vector of PiggyBac (PB) in addition with 2 μg transposase using LTX (Invitrogen). The cell selection was continued for seven days by using 2 μg/ml puromycin.

### Cell proliferation assay

The experiments of Cell Counting Kit-8 (CCK-8) was performed to assess the proliferation of HT-22 cells based on the manufacturer’s instructions. Cell culture *in vitro* was conducted in 96-well plates with 100 μl DMEM supplemented with 10% FBS. The cell density was set as 1000 cells per well. Three time points (24 hours, 36 hours, 48 hours and 60 hours after transfection) were selected for observation. The cell viability was determined based on the absorbance at 450 nm by using a microplate reader (Spectramax i3 platform, MD, Austria). All of the experiments were performed in three biological repeats.

### Experimental grouping and RNA sequencing

Two groups of the control (named as PB-1 and PB-2) and two groups of the treatment (named as Pias1-1 and Pias1-2) were selected in the following experiments. Total RNA in each group was extracted by using TRIzol, the cDNA was synthesized from 1 μg of total RNA by using reverse transcriptase (Takara). The preparation of the cDNA library and RNA sequencing (RNA-Seq) utilizing the platform of Illumina HiSeq 3000 were performed by Personal Biotechnology (Shanghai, China). On average, 6 G reads per sequencing sample were obtained. Based on the general process of data analysis, our sequencing data was analyzed [44]. The data preprocessing and quality control were performed by using the softwares of Trim Galore and FastQC. Using STAR software, the preprocessing sequence alignment was performed based on the mouse reference genome sequences (Mus_musculus.GRCm.38.83) downloaded from the database of Ensembl. Cufflinks software was used to perform transcript assembly and Cuffcompare software was employed to parsimoniously merge the assembled transfrags. The DEGs analysis was performed by using the package of DESeq2 under R software [45]. The significance threshold is defined as *p* < 0.05 and |log2 FC| > 1. The functional enrichment analysis was performed by GSEA to identify the significantly enriched KEGG pathways and GO terms, including biological process (BP), molecular function (MF) and cellular component (CC) [46]. The significance cutoff was chosen as FDR < 0.05. The web server SMART version 8.0 was employed to identify and annotate the specific domains of the target proteins [47]. The protein-protein interaction network was reconstructed by using the tool of STRING version 11.0 [48].

### Transcription factor binding site (TFBS) prediction

The promoter sequences, including 3000 bases upstream and 50 bases downstream of STAT1, were downloaded from the UCSC Genome Browser [49]. The animal transcription factor database AnimalTFDB 3.0 was employed to predict the transcription factor binding sites (TFBS), as well as predicting the associated TFs and their family information [50]. Next, we compared the above TFBS genes with the DEGs identified by RNA-seq data analysis to explore the overlapping genes that were affected by overexpressing PIAS1.

### Quantitative polymerase chain reaction (qPCR)

The primers of target genes were designed by using Primer 5.0. The experiments of qPCR were performed using TransStart Tip Green qPCR SuperMix (Takara). The normalization of each transcript expression was performed by using the 2−ΔΔCt method with the reference gene ribosomal protein L19 (Rpl19). The sequences of above primers are shown in Table 4.

**Table 4.** The primers of target genes for qPCR.

**Genes**	**Forward**	**Reverse**
Arnt2	GTTCCAGGACATGCTACCCAT	ATGCCCAGGTCAGCAAAGTC
Creb5	GTTGAAAAACGAGGTGGCCC	AGGGCTGCTCTCTGGACTTA
Dbp	CAAGCCCAAAGAACCGGC	AGCGGCGCAAAAAGACTCG
Etv1	TGCAAAAGGTGGCTGGAGAA	ATGTCCGTCTTCAGCAGTGG
Fosl1	GTTCCACCTTGTGCCAAGCA	GGACTGTACTGGGGATAGGC
Jun	CAAAACTCCGAGCTGGCATC	TGCGTTAGCATGAGTTGGCA
Klf6	CTTGAAAGCACATCAGCGCA	TCTTGCAAAACGCCACTCAC
Nr1h4	GAGATGGGGATGTTGGCTGA	CAGCGTGCTGCTTCACATTT
Nr4a1	CCGGTGACGTGCAACAATTT	TCCCCTCACCGGGTTTAGAT
Nr6a1	CATCCAGTAGGTCTGTGGAACT	TGGTGAGTGGCCAGAATAGC
Plau	TAAAATGCTGTGTGCTGCGG	CCGGGCTTGTTTTTCTCTGC
Tef	GAAGCTGATGGAGAACCCCC	GAAGGACTCGCCATCGTAGG
Egr1	AGTGATGAACGCAAGAGGCA	TAGCCACTGGGGATGGGTAA
Egr2	ATCGAAAGCCGTTTCCCTGT	GCGGATTATAAGGGGTGGCA
Egr3	GATGGCTACAGAGAATGTGATGG	TTGGAAGGAGAGTCGAAAGCG
Fos	TCGCATCAAGGCCATCATTG	TACGCACGTAGACCAGGATC
Tbr1	GGATTTACGAGCAGGCCAAG	CTATGTCCTTGGCGCAGTTC
Fabp4 (p15)	GGGATGGAAAGTCGACCACA	CTTGTGGAAGTCACGCCTTT
Stmn1 (p18)	TTCTCAGCCCTCGGTCAAAA	CTGCTTCAAGACTTCCGCCT
Cdkn1a (p21)	AGTACTTCCTCTGCCCTGCT	GAATCTTCAGGCCGCTCAGA
Cdkn1b (p27)	TCGCAAAACAAAAGGGCCAA	TTACGTCTGGCGTCGAAGG
Trp53 (p53)	GCCCATGCTACAGAGGAGTC	TCAGGCCCCACTTTCTTGAC
Coro1a (p57)	CTACCTCTGTGGCAAGGGTG	CCATACCACGCTGAGACTCC
Rpl19	GACGGAAGGGCAGGCATATG	TGTGGATGTGCTCCATGAGG

### Immunofluorescence staining

Cells were fixed in 4% paraformaldehyde (PFA fixative) for 20 mins at room temperature, washed in PBS three times, permeabilized for 1 h at 37°C in PBS-T solution(PBS containing 5% BSA and 0.2% Triton X-100), rinsed with PBS three times and incubated overnight at 4°C with the following primary antibodies: APP (25524-1-AP, Proteintech, 1:500). In dark, Alexa Fluor 594 (Invitrogen, 1:1000)-conjugated secondary antibody was used at 1:1000 and nuclei were stained with Hoechst (Invitrogen, 1:5000).

### Western blotting

Cells were lysed in pre-cooled RIPA cell buffer (P0013B, Beyotime Biotechnology, China) containing protease inhibitor (DI111-02, TRANSGEN BIOTECH, China, 1: 100). Proteins were separated by a 10% SDS-PAGE gel at RT and electrotransferred to a PVDF membrane in ice-bath. Probing was performed with specific primary antibodies and HRP-conjugated secondary antibodies. The primary antibodies used were PIAS1 (ab2474-1, Abcam, 1:1000) and GAPDH (SC-8035, Santa Cruz, 1:2000). Proteins were detected in super ECL detection reagent (No.180-501, Tanon, China).

## Supplementary Material

Supplementary Table 1

Supplementary Table 2
